# Supernatant of activated platelet-rich plasma rejuvenated aging-induced hyposalivation in mouse

**DOI:** 10.1038/s41598-023-46878-3

**Published:** 2023-12-01

**Authors:** Sungryeal Kim, Jeong Mi Kim, Eun Jeong Jeon, Ji Won Kim, Mi Eun Choi, Jin-Mi Park, Jeong-Seok Choi

**Affiliations:** 1 Department of Otorhinolaryngology-Head and Neck Surgery, Inha University College of Medicine, 27 Inhang-ro, Jung-gu, Incheon, 22332 Republic of Korea; 2 Department of Biomedical Science, Program in Biomedical Science and Engineering, Inha University, 100 Inharo, Michuholgu, Incheon, 22212 Republic of Korea; 3 Research Center for Controlling Intercellular Communication (RCIC), College of Medicine, Inha University, 100 Inharo, Michuholgu, Incheon, 22212 Republic of Korea

**Keywords:** Oral manifestations, Salivary gland diseases

## Abstract

Hyposalivation is a common complaint among the elderly, but no established treatment prevents age-induced hyposalivation. Platelet derivatives such as platelet-rich plasma (PRP), platelet-rich fibrin (PRF), and plasma rich in growth factor (PRGF), are used widely in different areas of regenerative medicine to enhance the wound healing processes. This study examined whether the local injection of the supernatant of activated PRP (saPRP) into the salivary gland (SG) could help prevent aging-induced SG dysfunction and explored the mechanisms responsible for the protective effects on the SG hypofunction. The platelets were separated from the blood of male SD rats (220 ± 20 g). saPRP was manufactured by removing the fibrin clot after activating platelet with calcium ionophore 10 μM (A23187). The total protein and TGF-β1 levels were significantly higher in saPRP than in PRP. Human salivary gland epithelial cell(hSGEC) was treated with saPRP or PRP after senescence through irradiation. The significant proliferation of hSGEC was observed in saPRP treated group compared to irradiation only group and irradiation + PRP group. Cellular senescence, apoptosis, and inflammation significantly reduced in saPRP group. The SG function and structural tissue remodeling by the saPRP were investigated with naturally aged mice. The mice were divided into three groups: 3 months old (3 M), 22 months old (22 M), and 22 months old treated with saPRP (22 M + saPRP). Salivary flow rate and lag time were significantly improved in 22 M + saPRP group compared to 22 M group. The histologic examinations showed the significant proliferation of acinar cell in 22 M + saPRP group. The decrease of senescence, apoptosis, and inflammation observed by western blot in 22 M + saPRP group. The saPRP induced the proliferation of hSGECs, leading to a significant decrease in cellular senescence via decrease inflammation and apoptosis, in vitro. Moreover, the acini cells of the salivary gland were regenerated, and the salivary function increased in aged mice. These results showed that saPRP could be a treatment agent against aging-induced SG dysfunction.

## Introduction

Saliva, synthesized and secreted by the salivary glands (SGs), plays an essential role in the oral cavity by maintaining oral homeostasis, protecting against infection, and promoting digestion. It consists of water (95%), inorganic salts and enzymes (0.2%) and proteins (0.3%)^1^. A dysfunction of the SG leads to xerostomia or dry mouth, sialadenitis or salivary gland inflammation, worsening of dental caries, and periodontal diseases. Furthermore, xerostomia reduces overall health or the quality of patient’s life^2^. Temporal xerostomia is caused by acute infection or dehydration^3^. On the other hand, permanent xerostomia is caused by autoimmune inflammatory diseases such as Sjogren syndrome, radiation therapy in head and neck cancer patients, xerogenic medication, or aging^4^.

The prevalence of xerostomia is approximately 20% in the general population, but it increases to 50% in older people^5,6^. Affoo et al. reported that the resting and stimulated salivary flow rate from the submandibular gland (SMG) and sublingual gland (SLG) and the overall resting salivary flow were significantly lower in older people, regardless of medication use by meta-analysis^7^. Previous studies reported decreased saliva secretion with increasing age because of the morphological changes in fat and fibrous tissues in human SG^8^. A study using aged animal SGs showed that decreased saliva production was associated with histological changes, acinar cell atrophy, cytoplasmic vacuolization, lymphocyte infiltration, and increased fibrotic tissue^9^. Miyagi et al.^10^ reported that cellular senescence, chronic inflammation, and decreased AQP5 expression are the likely causes of hyposalivation in the SGs of aged mice. Another cause of xerostomia is radiotherapy. In salivary glands irradiated with radiation, senescence occurs similar to the aging process, resulting in acinar cell loss^11,12^. There are many attempts are being made to prevent the damage or achieve acinar cell regeneration to treat the decrease in acinar cells caused by damage to the salivary gland, but there is no established treatment in xerostomia.

Platelet derivatives are biomaterials containing inactivated or activated platelets^13^. Activated platelet secretes various bioactive components such as chemokines, cytokines, growth factors (platelet derived growth factor, transforming growth factor β, and epithelial growth factor), and various proteins, including anti-aging proteins, such as tissue inhibitor of metalloproteinase 2 and growth differentiation factor 11^14^. There is increasing clinical interest in using platelet concentrates for local applications for dentistry^15^, dermatology^16^, ophthalmology^17^, orthopedics^18^, and plastic surgery^19^. In the clinical study, patients with head and neck cancer who suffered from post-radiation xerostomia showed significant improvement in all SG functions and life parameters through regeneration of acinar cell after treatment with modified platelet-rich plasma^20^.

The prevalence of chronic diseases related to aging is expected to increase, resulting in higher social costs because of the increased life expectancy of the aging population. Therefore, aging-induced SG dysfunction processes and new therapeutics developments in this field must be improved and encouraged. This study examined whether the local injection of supernatant of activated PRP (saPRP) into SGs could protect aging-induced SGs dysfunction, and explored the mechanisms responsible for the effects of saPRP on aging induced SG hypofunction. This study is the first to investigate the therapeutic application of saPRP to restore SG hypofunction in aged mice.

## Materials and methods

### Animals

Sprague–Dawley rats (250 ± 20 g) and C57BL/6 mice (four-week-old) were purchased from the Research Model Producing Centre (Orient Bio, Gyeonggido, Korea) for the study and fed a diet of animal chow and tap water and were housed at 20 ± 2 °C and humidity in a 12 h light–dark cycle according to the Guide of the Care and Use of Laboratory Animals by Inha University, Korea. The protocol used in this study was approved by the Animal Ethnics Committee of Inha University Hospital [INHA 190312-624, 170201-478].

### Blood collection and preparation of supernatant of activated platelet-rich plasma (saPRP)

Rat blood was drawn from the heart after surgery using a disposable syringe containing 2.2% sodium citrate (9:1, v/v). PRP was prepared by centrifuging citrated blood at 200 g for 10 min. The platelet number was adjusted to 4–4.5 × 10^8^/mL. Platelet activation agonist, 10 μM A23187 (Calcium Ionophore), was added. The reaction stopped at 20 min by cooling at 4 °C. The platelets released their therapeutic proteins into the surrounding plasma upon activation, and a fibrin clot was removed by centrifugation. A 3 kDa MWCO centrifugal filter unit (EMD Millipore, USA) was used to concentrate the protein in the solution. saPRP was aliquoted and stored at − 80 °C after use.

### Quantification of total protein, TGF-β1, and PDGF

When platelets are activated, many active proteins, cytokines and growth factors are released^21^. The total protein content was measured using a bicinchoninic acid (BCA) protein assay (Thermo Fisher Scientific, USA), transforming growth factor-beta 1 (TGF-β1), and platelet-derived growth factor (PDGF) concentrations were quantified using enzyme-linked immunosorbent assay (ELISA) (R&D systems, USA) according to the manufacturer’s instruction.

### Human salivary gland epithelial cells (hSGECs) culture

The hSGECs were obtained from a patient who underwent parotidectomy due to a benign parotid tumor. The specimens were collected with informed consent and institutional review board approval [INHA 180503-560]. A small portion of a non-tumor bearing gland was resected and washed with HBSS containing 1% antibiotics. The tissue was chopped with fine scissor and filtered through a 70 μm cell strainer, then centrifuged at 1500 rpm for 5 min, after which it was plated on a culture dish with Keratinocyte serum-free media (Gibco, USA) containing L-glutamine, 2.5 μg of EGF, 0.09 mM CaCl_2_, and 1% antibiotics.

### Irradiation-induced cellular senescence

A low radiation dose can induce cellular senescence in many cell types^2^. To investigate irradiation-induced cellular senescence, the hSGECs at passages 3–5 were seeded at 4 × 10^4^ cells/well, cultured at 37 °C for one day on eight well slide chamber (Corning Corp., USA) and irradiated with 15 Gy using a 4 MV X-ray from a linear accelerator (Mevatro MD, Siemens Medical Laboratories Inc., Germany). The saPRP for one day was administered immediately after irradiation. The cellular senescence was detected using a β-galactosidase staining kit (Sigma Aldrich, USA) according to the manufacturer’s instruction and analyzed under an optical microscope (Olympus FV1000, Olympus, Japan).

### Cell proliferation test

The Cell Counting Kit-8 (CCK-8, Dojindo, Japan) assay was used to investigate the cell proliferation of hSGECs. The hSGECs were seeded at 1 × 10^4^ cells/well and cultured at 37 °C for one day. The cultured cells were divided into four groups: (1) Normal control group without irradiation (Control), (2) PRP or saPRP treatment group, (3) Irradiation group (IR), and 4) PRP or saPRP treatment after irradiation group. The IR treatment was irradiated with 7 Gy using 4 MV X-rays from a linear accelerator. The PRP or saPRP group was exposed to PRP or saPRP in the culture media, and the IR + PRP or saPRP group was exposed to PRP or saPRP after irradiation. After adding of 10 μL of CCK8 reagent and incubation at 37 °C for three hours, the proliferation of hSGECs was investigated by reading the absorbance at 450 nm using a 96-well plate reader (Dynex Revelation, Dynex Ltd., UK).

### Preparation of cell block and terminal deoxynucleotidyl transferase biotin-dUDP nick and labeling (TUNEL) assay

The cell blocks were prepared using Histogel (Thermo Scientific, USA) for immunostaining. The cells were washed and fixed in 4% paraformaldehyde for 30 min at RT. After washing twice with PBS, the cells were loaded on pre-heated Histogel and incubated for 30 min at 4 °C. The histogel block was fixed in 10% formalin for two days and processed paraffin embedding. The apoptotic cells in cell blocks and submandibular glands were detected using an ApopTag Peroxidase In Situ Apoptosis Detection Kit (Roche Diagnostics, Canada). The sections were then incubated with anti-digoxigenin conjugate at RT for 30 min, after which the nuclei were detected using Mayer’s Hematoxylin. The TUNEL-positive apoptotic cells were counted in three random sites under an optical microscope (Olympus FV1000, Olympus, Japan).

### Amylase activity

The amylase activity of the hSGECs was determined using a salivary α-amylase assay kit (R&D system, USA) with 2-chloro-p-nitrophenol linked with maltotriose as the chromogenic substrate according to the manufacturer’s instructions. The α-amylase activity in the sample directly was proportional to the increases in absorbance at 405 nm observed using a 96-well plate reader.

### Supernatant treatment in aged mice

Three- and 22-month-old female C57BL/6 mice were purchased from the Research Model Producing Centre (Orient Bio, Gyeonggido, Korea) for the study. The animals were housed in a temperature, humidity, and light-controlled environment on a standard mouse diet with free access to tap water. The mice were divided into three groups: Young group (3-month-old mouse with only neck incision; n = 5), Old group (22 months old mouse with only neck incision; n = 5), and saPRP group (22 months old mouse with supernatant injection; n = 5). The mice in the saPRP groups received an injection of saPRP into the submandibular gland. Briefly, a neck incision was performed to expose the submandibular glands, and the 40 µl of saPRP was then injected directly into both glands using a syringe with a 25-gauge needle.

### Measurements of body weights, salivary gland weights, salivary lag times, and salivary flow rates

The body weights were measured four weeks after the supernatant treatment and saliva was collected from the mouth floors using a micropipette 10 min after muscarinic cholinergic agonist pilocarpine (0.2 mg/kg i.p.). Salivary flow rates (SFRs) and lag times were measured at four weeks after the supernatant treatment. The salivary lag times were calculated from salivary stimulation to the beginning of saliva secretion. The mice were euthanized four weeks after supernatant treatment. The submandibular glands were harvested, surrounding fat and connective tissues were removed with a dissecting microscope and the weights of both submandibular glands in each animal were measured.

### Histological analysis and immunohistochemistry

For histological analysis, the dissected submandibular gland tissues were fixed in a 4% paraformaldehyde phosphate buffer solution for 48 h before being embedded in paraffin. A 5 μm thick section was dewaxed and hydrated, stained with hematoxylin & eosin (H&E) and Masson’s trichrome (MT) and examined under a digital microscope (Olympus, Japan). The immunohistochemical study used the following antibodies: Aquaporin 5 (AQP5; 1:200; Calbiochem, USA). A blinded examiner evaluated three random fields per section, and stained areas were measured in pixels using the Image J software (MD Anderson Center, USA).

### RNA isolation and analysis

The total RNA was isolated using the RNeasy Mini kit (Qiagen, Germany). The complementary DNA (cDNA) was synthesized form the total RNA using the Tetro cDNA synthesis kit (Bioline, USA). The reaction mixture was incubated at 45 °C for 30 min, heated to 85 °C for 5 min, and cooled to 4 °C. The cDNA was used as the template to perform, a real-time polymerase chain reaction (RT-PCR) in a 96-well plates (Applied Biosystems, USA) using the SYBR green II Master Mix (Takara Bio Inc., Japan) in StepOne (Applied Biosystems, USA) with the following thermal cycle: 95 °C for 20 s and 40 cycles of 95 °C for 5 s and 60 °C for 20 s. AQP5, interleukin 6 (IL6), p16^INK41^, p21, Bax, and Bcl_xl_ were amplified; β-actin was used as the endogenous control.

### Statistical analysis

Statistical analysis was performed using the GraphPad Prism 8 package (GraphPad Software Inc, La Jolla, CA). Comparisons among four groups were performed using one-way ANOVA followed by a Tukey’s post hoc test, and paired group comparisons were performed using two-way ANOVA followed by a Bonferroni post hoc test at a significance level of *p* < 0.05. The results are expressed as the mean ± standard deviation. All experiments were performed using at least four different mice for each group, with “n” referring to the number of animals.

## Results

### Release of proteins upon platelet activation

**Figure 1:**
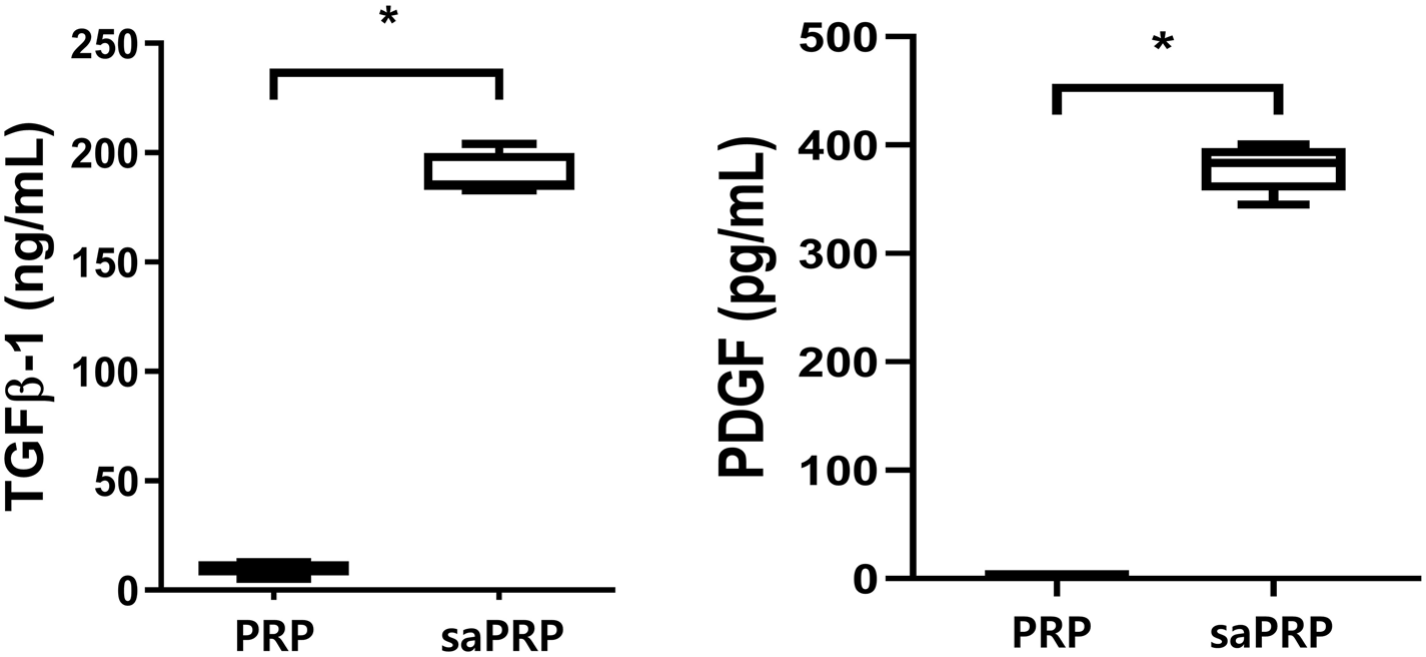
Concentration of transforming growth factor β-1 (TGF β -1) and platelet-derived growth factor (PDGF). PRP: platelet-rich plasma. *saPRP* supernatant of activated platelet-rich plasma. *: *p* < 0.05.

When the platelets were activated, several growth factors, including PDGF and TGF- β1 were secreted^13^. The concentration of therapeutic factors secreted upon platelet activation by the agonists (A23187) was initially analyzed. The total protein, TGF-β1, and PDGF concentrations were significantly higher in the saPRP than the PRP (Fig. 1). Platelet activation appeared to have been achieved considering growth factor concentrations.

### The saPRP treatment assists hSGECs in proliferating and escaping from senescence with or without irradiation

**Figure 2:**
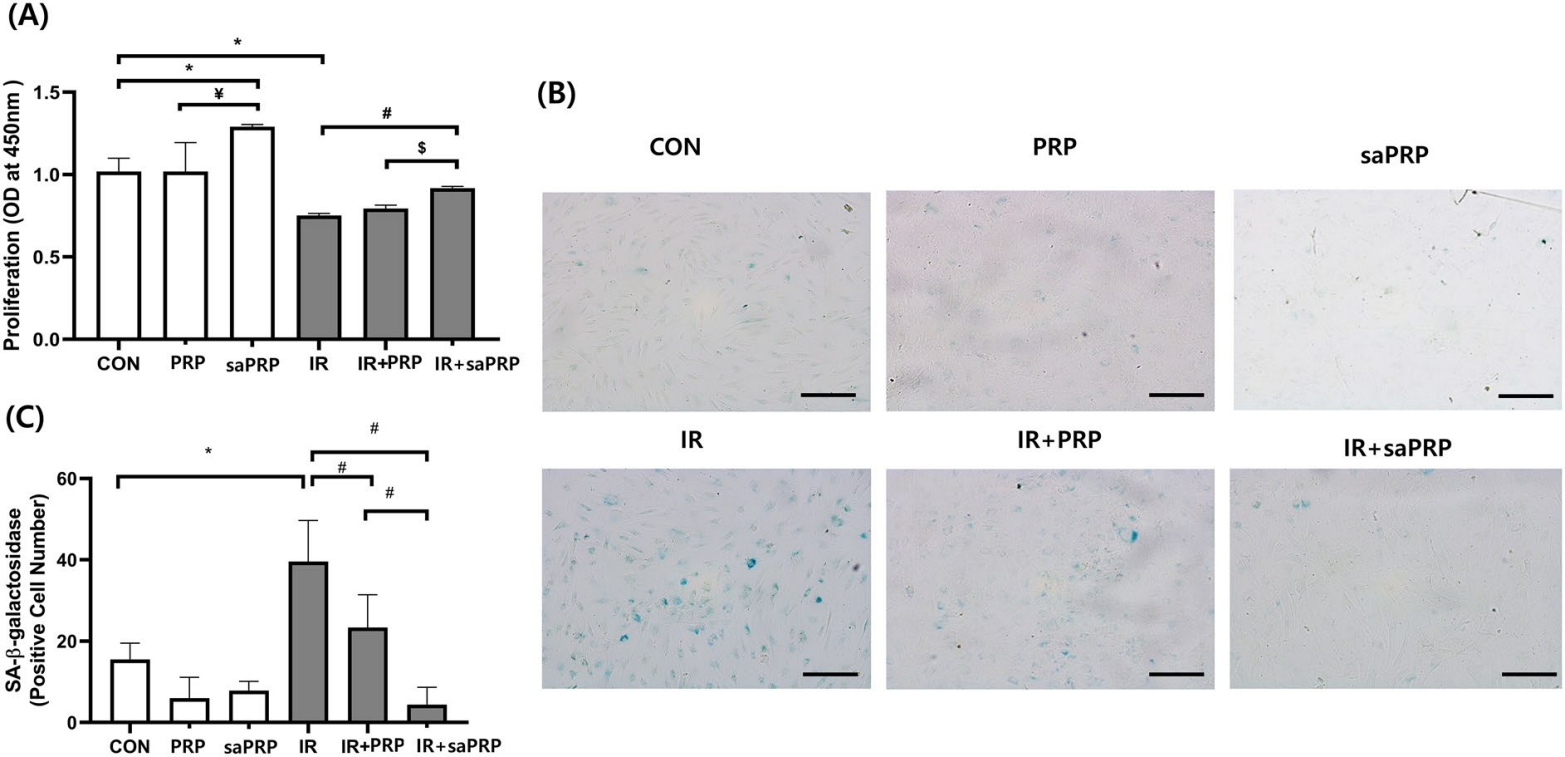
Anti-aging effect of irradiation and platelet derivatives on human primary salivary gland epithelial cells (hSGECs). (**A**) Proliferation (**B**) Microscopic view of β-galactosidase stating (**C**) Statistic analysis of β-galactosidase staining. *CON* control. *PRP* platelet-rich plasma. *saPRP* supernatant of activated platelet-rich plasma. *IR* irradiation. *: *p* < 0.001, compared to CON, ^¥^: *p* < 0.05, compared to PRP, ^#^: *p* < 0.01, compared to IR, ^$^: *p* < 0.05, compared to IR + PRP. Bar size: 100 μm.

A CCK8 assay was performed to evaluate the effects of PRP and saPRP on the proliferation of hSGECs (Fig. 2A). The duration of saPRP treatment was one day for all tested in vitro assays. In the case of not receiving radiation, PRP did not affect the proliferation of hSGECs compared to the control, but saPRP had a significantly positive effect on the proliferation of hSGECs. Subsequently, when PRP or saPRP was administered after radiation, the IR + saPRP group showed a significantly higher proliferation of hSGECs compared to the IR and IR + PRP groups.

SA-β-gal SA-β-gal is a well-known marker of aging. The senescence model induced by irradiation at 7 Gy in hSGECs was used to explore the role of PRP or saPRP in cell senescence. Figure 2B shows the results of SA-β-gal staining. The senescence of hSGECs was accelerated by 7 Gy radiation. Both PRP and saPRP reduced SA-β-gal expression regardless of irradiation groups. On the other hand, the saPRP treatment significantly relieved the degree of senescence in IR-induced aged hSGECs rather than PRP treatment (Fig. 2C). Based on the above results, subsequent experiments were conducted with only saPRP.

### Preventive effect of saPRP in cellular senescence of SGECs

**Figure 3:**
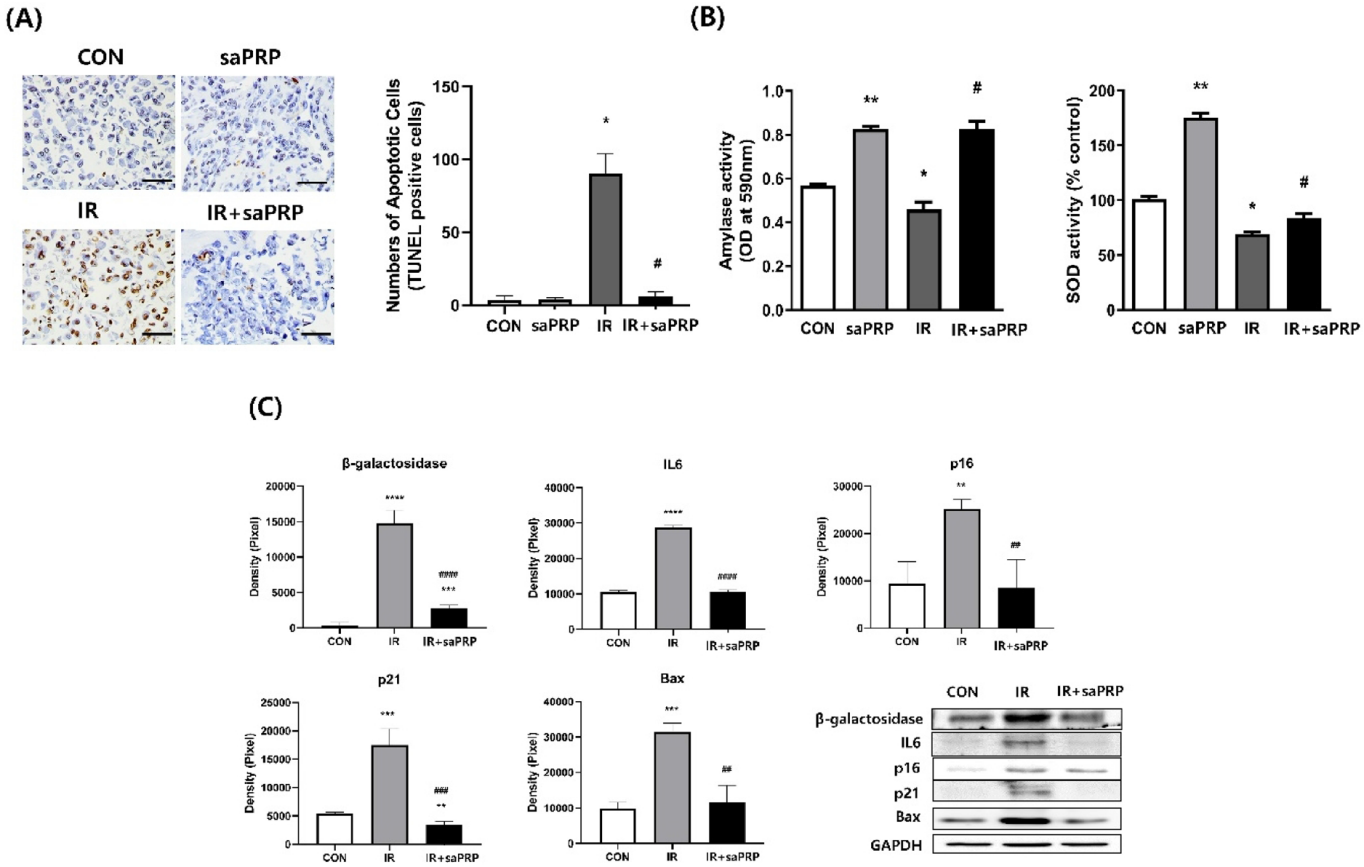
Protective effect of saPRP on irradiation in human primary salivary gland epithelial cells (hSGECs). (**A**) Terminal Deoxynucleotidyl Transferase Biotin-dUDP nick and labeling (TUNEL) assay. (**B**) Amylase and superoxide dismutase (SOD). (**C**) Western blot. *CON* control. *saPRP* supernatant of activated platelet-rich plasma. *IR* irradiation. Uncropped results of western blot were shown in supplementary file (Figure S1 and S2). *: *p* < 0.05, **: *p* < 0.01, ***: *p* < 0.001, *****: *p* < 0.0001 compared to CON, ^#^: *p* < 0.05, ^##^: *p* < 0.01, ^###^: *p* < 0.001 compared to IR. Bar size: 100 μm.

Among the changes caused by irradiation-induced senescence, DNA damage induces aging and can cause apoptosis^22^. Platelet derivatives have been reported to reduce irradiation-induced apoptosis^23^. The anti-apoptotic effect of saPRP was investigated by TUNEL assay. In the IR-induced aging model, saPRP treatment reduced apoptosis, which was statistically significant (Fig. 3A). Amylase and SOD decreased in the aged hSGECs. When the hSGECs were treated with saPRP, the amylase and SOD levels quantified by ELISA increased significantly in the senescence and control models (Fig. 3B). Western blot analysis showed the aging-related factors (SA-β-gal, p16, and p21), the inflammatory factor (IL-6), and the apoptosis-related factor (Bax) increased significantly in the IR group compared to the control group, and all factors were significantly lower in the saPRP -treated group than the IR group (Fig. 3C). Based on the experimental results, saPRP appears to alleviate the aging-related dysfunction of hSGECs by reducing DNA damage-induced apoptosis and cellular senescence.

### saPRP treatment preserved the structure and function of submandibular gland in aged mice

**Figure 4:**
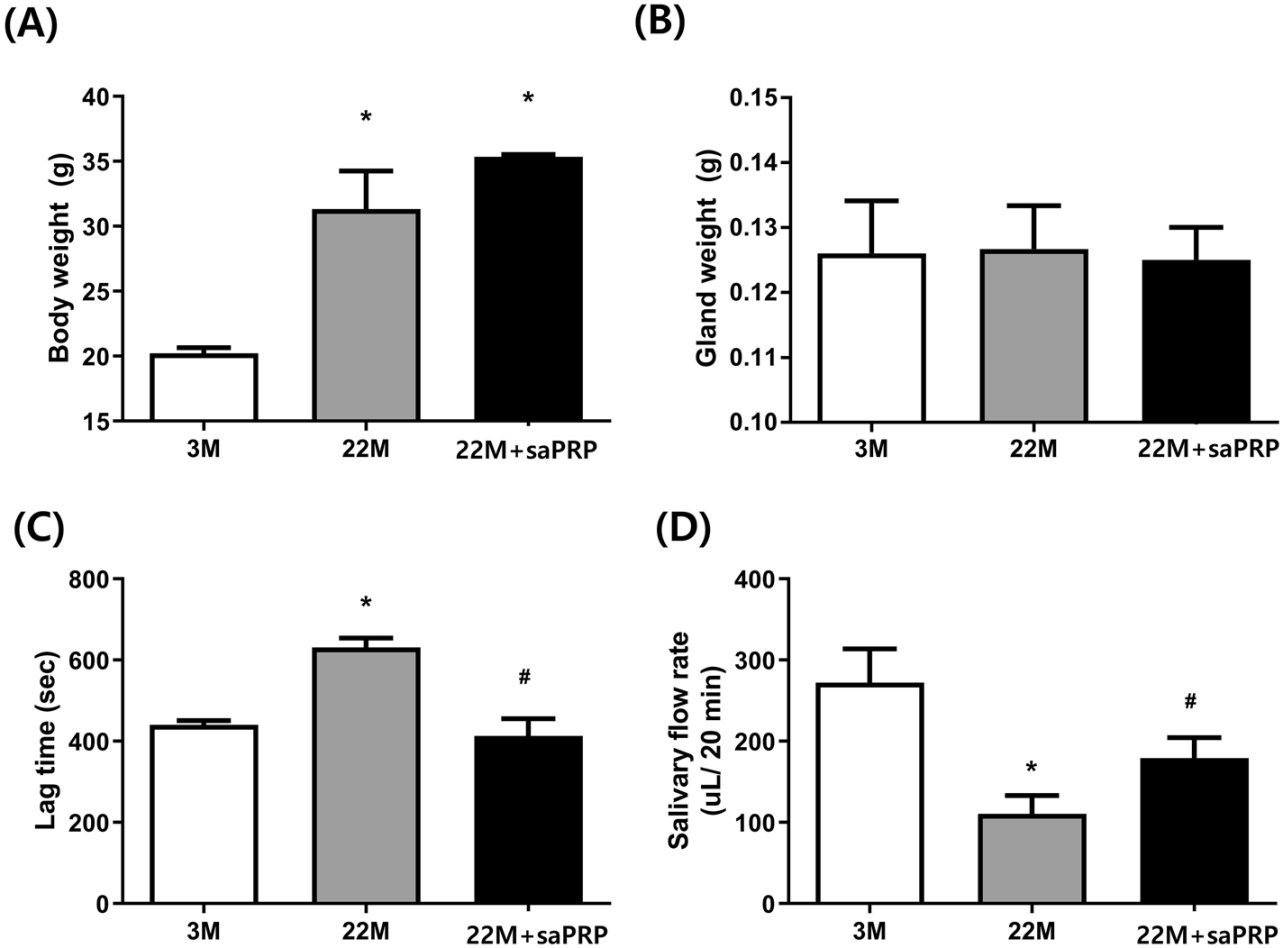
Functional effect of saPRP on aged mice. (**A**) Body weight. (**B**) Gland weight. (**C**) Lag time. (**D**) Salivary flow rate. 3 M: 3 months old. *22 M* 22 months old. *saPRP* supernatant of activated platelet-rich plasma. *: *p* < 0.05 compared to 3 M, ^#^: *p* < 0.05 compared to 22 M.

**Figure 5:**
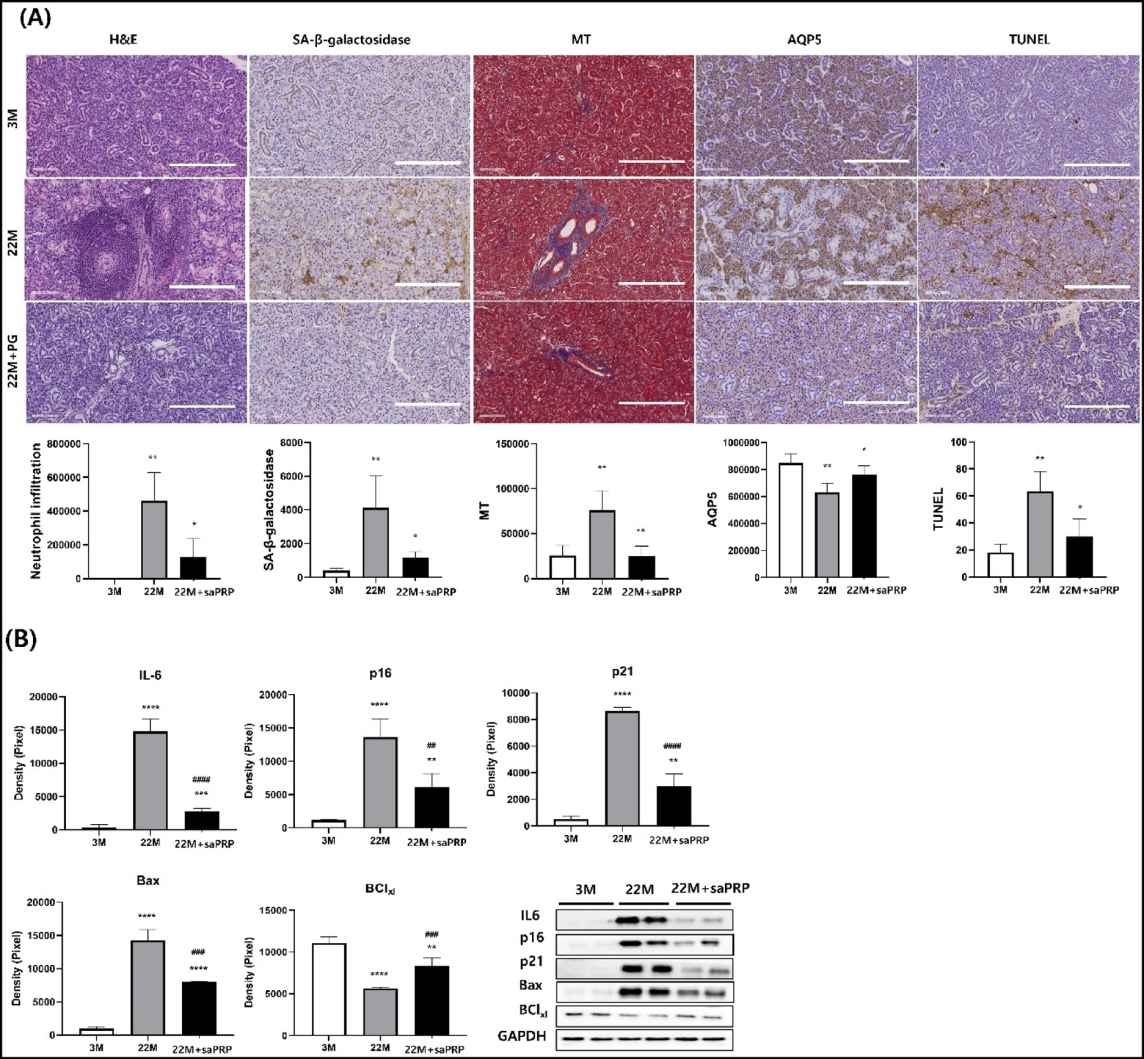
Changes after saPRP treatment of aged mice. (**A**) Histologic analysis. (**B**) Western blot. *H&E* Hematoxylin and eosin. *MT* Masson's trichrome. *AQP5* Aquaporin 5. *TUNEL* Terminal Deoxynucleotidyl Transferase Biotin-dUDP nick and labeling. *3 M* 3 months old. *22 M* 22 months old. *saPRP* supernatant of activated platelet-rich plasma. *: *p* < 0.05, **: *p* < 0.01, *****: *p* < 0.0001 compared to 3 M, ^##^: *p* < 0.01, ^###^: *p* < 0.001, ^####^: *p* < 0.0001 compared to 22MIR. Bar size: 500 μm.

After a neck incision, 40 µl of saPRP was injected directly into the submandibular gland of 22-month-old female mice (22 M + saPRP) to determine the effects of saPRP on aging. Aging of the salivary glands was confirmed using three-month-old (3 M) and 22-month-old (22 M) female mice as the control groups. The body weight and gland weights were similar between the 22 M and 22 M + saPRP groups (Fig. 4A, B). The lag time and salivary flow rate were significantly better in the 22 M + saPRP group than in the 22 M group (Fig. 4C, D). Histologic analysis was performed one month after a neck incision in all mice (Fig. 5). H&E staining showed that the salivary gland structure was observed in the 22 M + saPRP group. In contrast the lobular structure of the salivary gland was disrupted in the 22 M group compared with the 3 M group. Moreover, the leukocyte infiltration increased significantly in the 22 M group. SA-beta-gal expression was high in the order of 22 M, 22 M + saPRP, and 3 M group. The aged salivary gland showed increased periductal and perivascular fibrosis^9^. MT staining confirmed that periductal and perivascular fibrosis occurred the most in the 22 M group. AQP5 is a protein involved in the saliva secretion of acini cells and is used to evaluate its function^24^. AQP5 expression decreased less in the 22 M + saPRP group than in the 22 M group. The TUNEL assay showed that apoptosis was reduced in the 22 M + PG group, which is consistent with the in vitro experiment. IL-6, p16, p21, and Bax expression were confirmed by western blot; all four were significantly lower in the 22 M + saPRP group compared than the 22 M group.

## Discussion

Various growth factors and cytokines exist in the granule of platelet. When the platelet is activated, these growth factors and cytokines are secreted and involved in hemostasis, thrombosis, and immunoregulation^25^. The platelet plays a vital role by secreting the molecules necessary for each stage of wound healing (hemostasis, inflammation, proliferation and remodeling), so it is effective in wound healing and tissue regeneration such as oroantral fistula, diabetic foot ulcer, muscle injury, and tendonitis^26–29^. Moreover, a PRP injection affects skin rejuvenation, so it can be used for anti-aging purpose^30^. Platelet derivatives are utilized in an inactivated or activated state of platelet, depending on the manufacturing method. PRP containing inactivated platelets are obtained by centrifuging after collecting blood in a tube with an anticoagulant. When an activated platelet is desired, there are two methods, adding activating factors on PRP or collecting blood into a tube without anticoagulant. The method using activated platelets is expected to have a more significant effect because it contains more growth factors, but additional processes (activation process) and insoluble fibrin networks are formed^21,31^. The fibrin network caused by platelet activation is favorable to cell migration and retaining small molecules, such as growth factors, making it a good treatment for wound healing with defects. On the other hand, it is difficult to use in an injection due to insolubility. Herein, the supernatant was obtained after the activating PRP to make an injectable product rich in growth factors. This study assessed its potential as a treatment for age-induced salivary gland dysfunction.

There were many growth factors in the saPRP used in this study. Platelet-rich fibrin, plasma rich in growth factor, concentrated growth factor, and platelet lysate are typical methods using activated platelets. The fibrin network is formed in these product, which acts as a 3D scaffold to help cell migration and contains small molecule with controlled release^32^. An attempt was made to remove the fibrin network because the fibrin network is difficult to manipulate owing to its insolubility, but there was concern that growth factors would disappear due to entrapment within the fibrin network. On the other hand, Xiao et al. reported a regenerative effect even when the fibrin network was removed after platelet activation^33^. The saPRP had high concentrations of growth factors. This utilization is believed to expand the application of platelet derivatives by injecting them into deep tissues or using them in areas without tissue defects. Another advantage of removing fibrin network is reducing immune reaction. Among the components of platelet derivatives, leukocyte and platelet are directly and indirectly involved in immune reaction^34^. They are trapped by fibrin network after activation of platelet^35^. In our study, the fibrin network was removed to create an injectable form, and it is believed that leukocytes and platelets were removed as a result. saPRP will be a good alternative where immune reaction is not needed.

In the in vitro model, saPRP showed a more significant anti-aging effect than PRP. As mentioned above, the regenerative efficacy of platelet derivatives was clinically verified, and analgesic and anti-aging properties were also studied^34^. Regarding the anti-aging effect, Jia et al. reported PRP increased proliferation and decreased SA-β-gal staining in ultraviolet irradiation-induced aged mouse dermal fibroblasts^36^. Aging was ameliorated by PRP acting as an antioxidant. Platelet derivatives also affected on aged adipose tissue and bone marrow-derived stem cells^37^. They also suggested that oxidative stress was reduced by PRP, and SIRT1 increased, resulting in an anti-aging effect. As Li et al. suggested the NF-κB pathway as the anti-aging mechanism of platelet derivatives^38^. Reactive oxygen species activates NF-κB through IκB or IKK, an upstream of NF-κB, resulting in the expression of the pro-inflammatory cytokine interleukin-6 (IL-6)^39^. Several growth factors in platelet granules showed an antioxidant effect^40–42^. The platelet derivatives using activated platelet contain higher concentration of growth factors than PRP^43^. Therefore, it is not surprising that activated platelet derivatives proliferated cells better than PRP^44,45^. The study results revealed, a decrease in IL-6 when treated with saPRP, suggesting that the anti-aging effect was caused by the antioxidant action of the molecule secreted by platelet activation.

saPRP increased the proliferation of acini cells in the aged salivary gland and recovered the salivation of the salivary gland. This is the first in vivo result in which platelet derivative showed rejuvenation effect other than in the skin. The PRP injection is already clinically applied to the skin, and the seveal systemic reviews have confirmed the rejuvenation effect on the skin^30,46^. The results that platelet derivatives induced rejuvenation in other tissues was found only in in vitro models^37, 38^. Several methods have been attempted to address salivary gland dysfunction due to aging. Among them, stem cell implantation, delivery of bioactive compounds, and gene therapy have been studied to overcome the dysfunction through salivary gland regeneration^47^. The saPRP used in this study is a cocktail of growth factors. Therefore, it can be a method using a bioactive compound. Patients can use their blood, and saPRP can be considered superior to stem cell implantation and gene therapy in terms of stability because it is a small molecule. If the PRP preparation can be standardized, saPRP can be applied clinically faster than other treatments because platelet derivatives are already used clinically^48–50^.

In conclusion, the supernatant of activated PRP induced rejuvenation by proliferating acini cells of the salivary gland in naturally aged mice. These results show that bioactive compounds can induce regeneration and rejuvenation in the aged salivary gland. The potential of platelet derivatives as a source of bioactive compounds was also confirmed. Future studies standardizing the preparation and activation of platelet derivatives and confirm the mechanism of tissue regeneration and rejuvenation will be needed to confirm whether saPRP is a promising method for tissue regeneration and rejuvenation.

### Supplementary Information

Supplementary Figure S1.

Supplementary Figure S2.

## Data Availability

The datasets generated and/or analyzed during the current study are not publicly available due to containing personally identifiable information but are available from the corresponding author on reasonable request.
