# Supplementary figures and images for: Supernatant of activated platelet-rich plasma rejuvenated aging-induced hyposalivation in mouse
Source: Sci Rep. 2023 Dec 1;13:21242. doi: 10.1038/s41598-023-46878-3 (PMC10692196; doi:10.1038/s41598-023-46878-3)

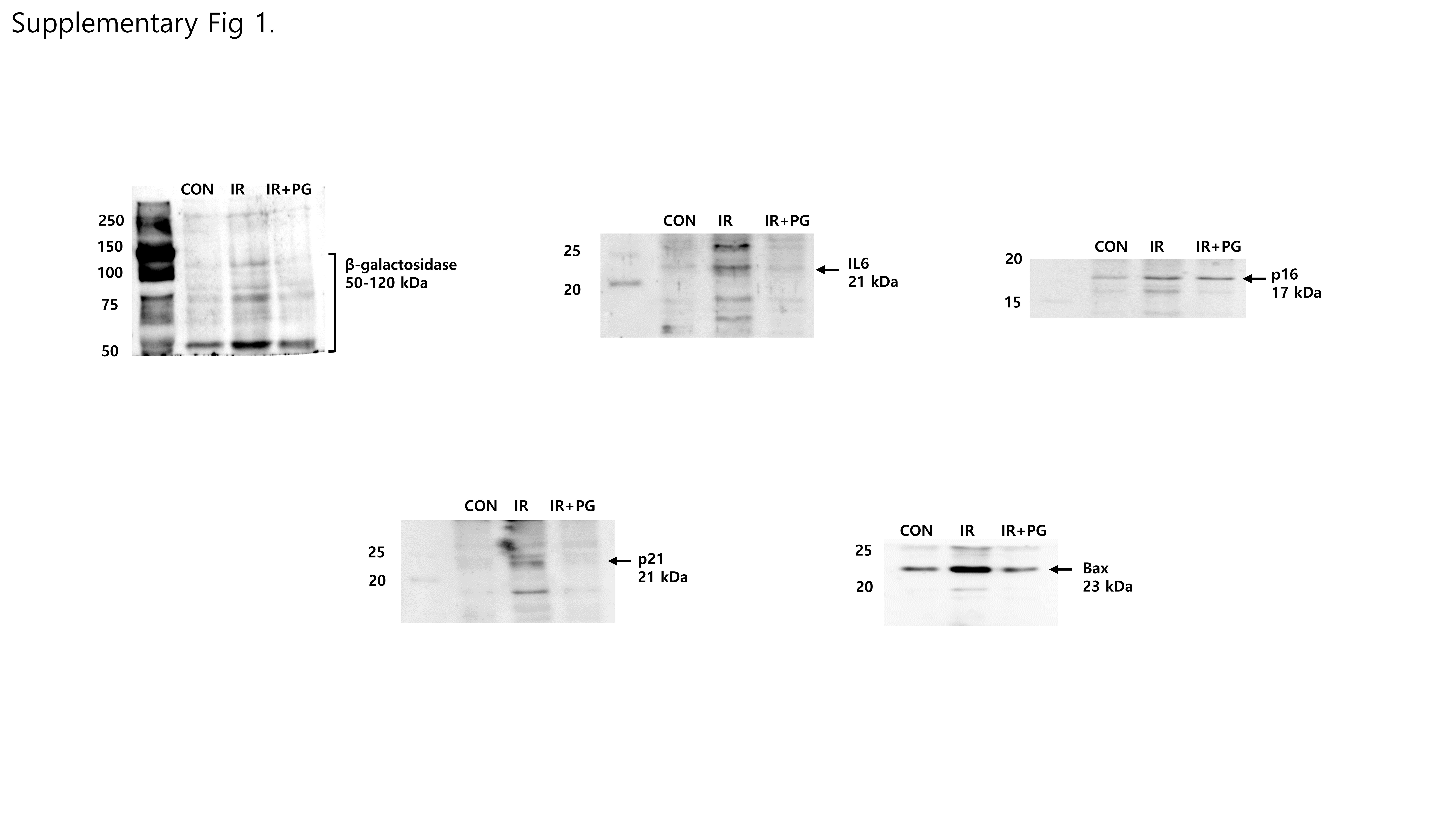

Supplement: Supplementary file 1 — Supplementary Figure S1. [file 41598_2023_46878_MOESM1_ESM.jpg]

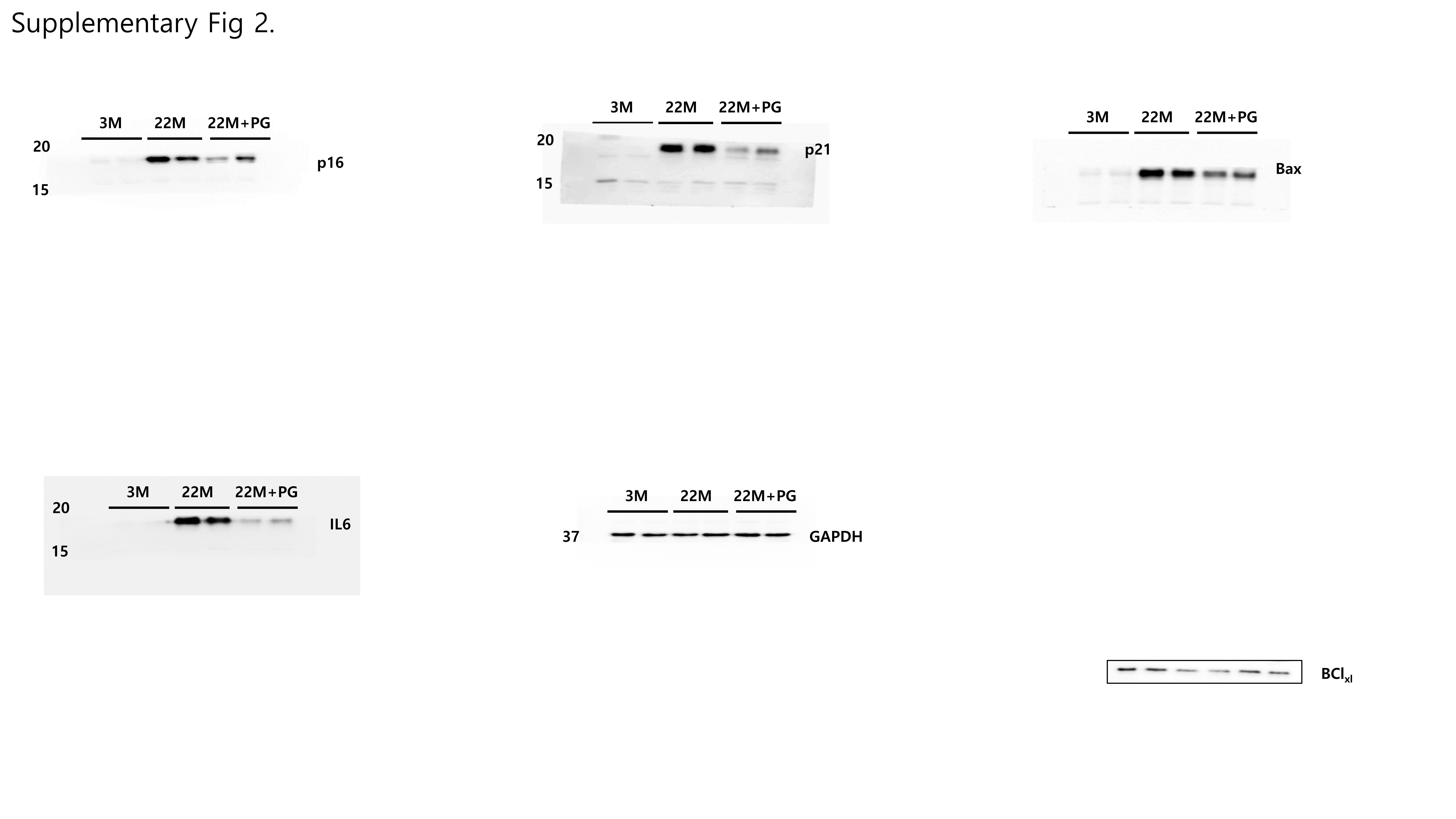

Supplement: Supplementary file 2 — Supplementary Figure S2. [file 41598_2023_46878_MOESM2_ESM.jpg]
